# Urdu adaptation and validation of a disease-specific quality-of-life questionnaire in a Pakistani population of Achalasia

**DOI:** 10.1371/journal.pone.0321933

**Published:** 2025-04-17

**Authors:** Sameen Abbas, Syed Sikandar Shah, Tayyab Saeed Akhhtar, Kiran Hameed, Saima Mushtaq, Amjad Khan

**Affiliations:** 1 Department of Pharmacy, Quaid-i-Azam University, Islamabad, Pakistan; 2 Department of Clinical Pharmacy and Pharmacology, RAK College of Pharmacy, RAK Medical and Health Sciences University, Ras Al Khaimah, UAE; 3 Center for Liver and Digestive Diseases, Holy Family Hospital, Rawalpindi, Pakistan; 4 Department of Pharmacy, The First Affiliated Hospital, Xi’an Jiaotong University, Xi’an, China; 5 Department of Pharmacy Administration and Clinical Pharmacy, School of Pharmacy, Health Science Center, Xi’an Jiaotong University, Xi’an, China; Riphah International University, Pakistan

## Abstract

**Background:**

Achalasia, a rare esophageal disease marked by dysphagia, impacts health-related quality-of-life (HRQoL), measurable by disease-specific tools like achalasia-specific questionnaire (ASQ) that assess symptom severity and QoL. However, to ensure its reliability and validity across different populations, cross-cultural adaptation is necessary. So, for this reason, this study aimed to validate an Urdu-translated version of an ASQ in a Pakistani population of achalasia patients.

**Methodology:**

A prospective cross-sectional study involving 52 participants was conducted at the Center for Liver and Digestive Diseases, Holy Family Hospital, Rawalpindi. ASQ was translated into the Urdu language by a forward-backwards translation process with expert input. Validation included factor analysis, known-group techniques, Cronbach’s alpha for reliability, and an independent t-test comparing ASQ scores with Eckardt scores for criterion validity.

**Results:**

Among 52 participants (27 males, median age 30 years; 25 females, median age 48 years), 63.5% had Achalasia type I, 26.9% type II, and 9.6% type III. Factor analysis confirmed a well-defined construct with good validity, and internal consistency was strong (Cronbach’s alpha = 0.89). The ASQ scores significantly correlated with Eckardt scores (p < 0.05), confirming its validity. 73.1% of participants found the translated version easy and completed in a short time duration.

**Conclusion:**

Urdu-translated ASQ proved to have good psychometric properties, with strong evidence of validity, reliability, and feasibility regarding health status in Pakistani achalasia patients. It can be recommended as a reliable QoL measure for clinical and research purposes. Future studies should explore its application in larger, more diverse cohorts and further refine its use in achalasia management.

## Introduction

Achalasia is a rare disease caused by the loss of ganglionic cells in the esophagus, leading to the denervation of the smooth muscles. It affects approximately 1–3 individuals per 100,000 and is mostly diagnosed in people aged 25–60. The condition is characterized by esophageal aperistalsis (lack of coordinated contractions) and impaired relaxation of the lower esophageal sphincter (LES) during swallowing, resulting in obstruction and dilation of the esophagus. Symptoms include difficulty swallowing (dysphagia), regurgitation, chest pain, and weight loss [1]. Diagnosis is typically made using a barium swallow X-ray to assess esophageal dilation and poor emptying, as well as esophageal manometry to measure LES relaxation and peristalsis and is classified into three subtypes: classic achalasia, achalasia with compression, and spastic achalasia, based on the pattern of pressure in the non-peristaltic esophagus [1,2].

Understanding quality-of-life (QoL) in achalasia requires a comprehensive framework that accounts for both symptom severity and its broader psychosocial consequences. The World Health Organization’s Quality of Life (WHOQOL) framework and the biopsychosocial model provide valuable perspectives, emphasizing the interplay between biological dysfunction, emotional distress, and social limitations. This approach is particularly relevant for moderate to severe medical conditions, as it assesses how the disease progression and related interventions impact daily functioning. Patient-focused questionnaires are commonly used to measure QoL, serving purposes such as validating diagnostic procedures, monitoring patient improvement, evaluating the effectiveness of treatments, and guiding clinical interventions [3,4]. In the case of achalasia, the Eckardt score and the achalasia-specific quality of life questionnaire (achalasia-DSQoL questionnaire and/or ASQ) are widely utilized to assess the quality-of-life (QoL) in patients with achalasia.

Eckardt score was first used and implied in the early 1990s and was based on the clinical practices of the researchers. It is a simple questionnaire that is widely employed for estimating QoL in patients of achalasia [5]. ASQ questionnaire is a straightforward, easy-to-use tool used for assessing clinical manifestations of achalasia, designed by Urbach and his colleagues, and is a validated ten items-based questionnaire where quantification and qualifications of esophageal symptoms of achalasia patients were made possible relating to dysphagia of solids as well as liquids, specific types of foods, other related symptoms, and overall QoL and/or health of patients of achalasia [6]. Its simplicity allows for practical application in clinical and research settings, making it accessible for both patients and healthcare providers. As achalasia is a rare disease with a very low prevalence, Urbach et al. were not able to validate their questionnaire in a population other than the one for which it was designed.

Cultural diversity can influence the applicability of health-related questionnaires [7] like ASQ. To ensure validity across different populations, the Food and Drug Administration (FDA) recommends validating the health status reported by patients in terms of their ethnic and linguistic differences, especially when applying the questionnaire to a new or different population [8]. Cross-cultural adaptation addresses the challenges related to cultural and linguistic differences and involves adapting and comparing the questionnaire to different ethnicities based on its source with that of the target population [9]. Additionally, validating the questionnaire in the local language not only improves communication between patients and healthcare professionals but also enables the assessment of QoL based on patients’ experiences without intervention from healthcare professionals [10,11].

While similar tools exist in other languages, no validated version tailored to the Pakistani demographic has been available. By refining the ASQ for local use, the study enhances diagnostic accuracy and symptom tracking to overcome cultural and linguistic barriers, allowing for a detailed assessment of disease symptoms and impaired QoL among Pakistani achalasia patients. In clinical practices and further research, ASQ needs to be translated and validated in the local or native language, Urdu, the local language of Pakistan, spoken by approximately 85% of the population. The current study aims to validate a translated version of the ASQ by evaluating the structural validity and reliability in Achalasia patients present in the population of Pakistan.

## Methodology

A prospective cross-sectional study was conducted at the Center for Liver and Digestive Diseases, Holy Family Hospital, Rawalpindi, from 7^th^ September 2022–15^th^ November 2023. The study received ethical approval from the Institutional Research and Ethics Forum of Holy Family Hospital, Rawalpindi, with protocol approval number 269/IREF/RMU/2022, and the Institutional Ethical Review Board and Bio-Ethical Committee (BEC) of Quaid-I-Azam University, Islamabad, with protocol approval number BEC-FBS-QAU2022–381. The sample size for validating the ASQ questionnaire was determined to be 52 participants, considering five participants for each item in the instrument and accounting for a 20% dropout rate [12]. Written informed consent was obtained from all participants.

### Translation

**Fig 1:**
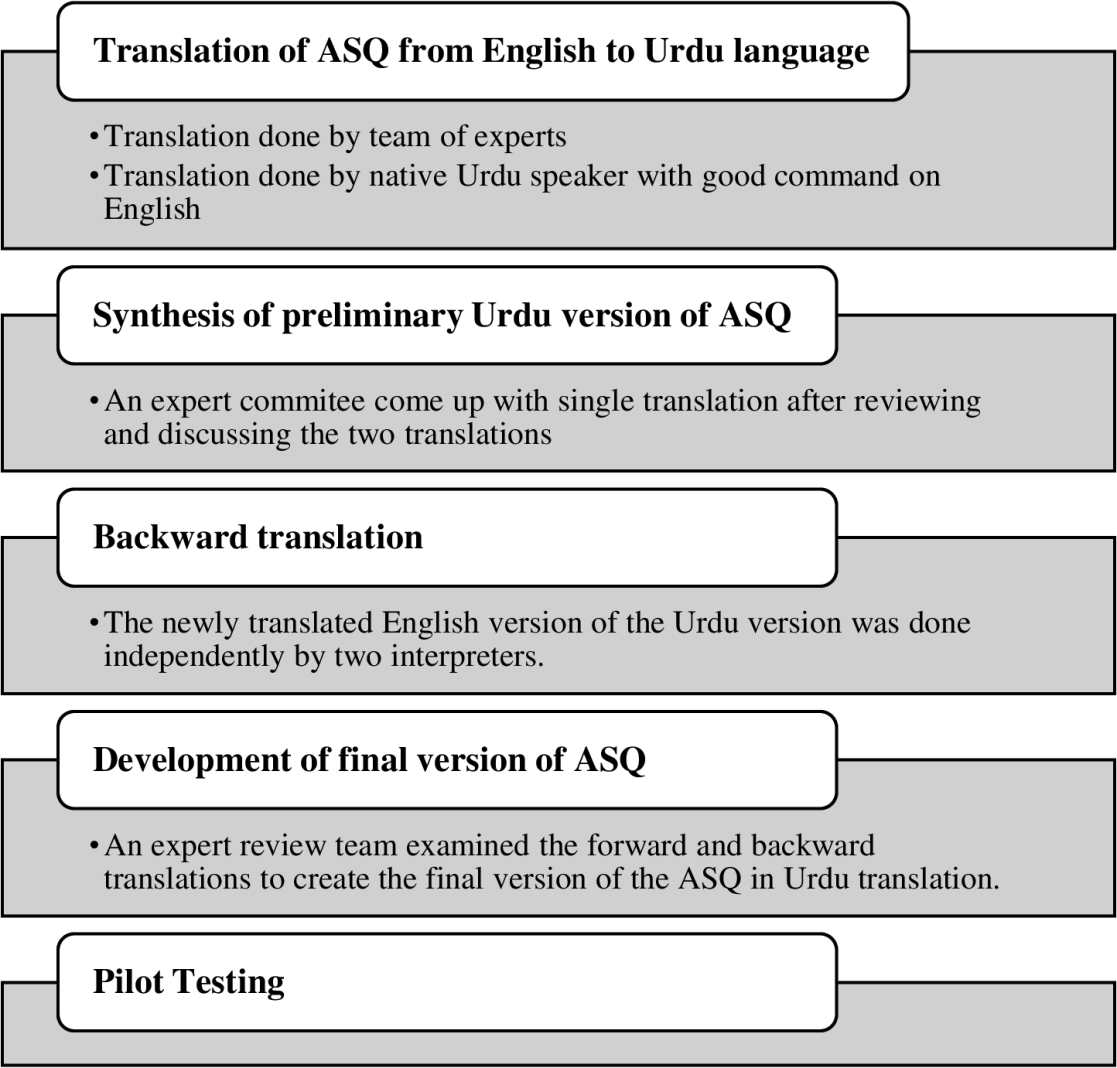
Translation and linguistic validation methodology used for translation of the ASQ. *Achalasia-specific questionnaire.

The Canadian English version of the ASQ questionnaire was obtained from previous literature (S1 Table) and made freely accessible. The translation into Urdu was performed by a team consisting of two research scholars and an assistant professor from the university. To ensure the accuracy of the translation, a native Urdu speaker proficient in English was chosen to forward-translate the ASQ from English to Urdu. The translated versions of the ASQ in Urdu were reviewed by a team of medical experts, academicians, and the translator, who discussed and resolved any discrepancies. To validate the newly translated Urdu version and ensure its content equivalence to the original version, two independent translators translated it back into English. The forward and backward translated versions, along with the input from the team of experts, including health professionals, academicians, and translators, were carefully reviewed to create the final version of the Urdu-translated ASQ. The translated version was critically reviewed for semantic, fluent, practical, and conceptual equivalence. Clear and culturally appropriate language was used in the translated questionnaire to reduce communication bias. To further validate the translated version, it was initially filled out by five achalasia patients, who were interviewed to ensure their understanding of the questionnaire and the meaning of each item. The patients reported no difficulty in comprehending or answering the questions. Therefore, the Urdu version of the ASQ was considered the final version of the questionnaire (Fig 1). The corresponding author can provide the Urdu version of the ASQ upon request.

### Study design

Patients were included if they: (1) had confirmed achalasia, (2) showed no other gastric disorders on endoscopy, (3) received no treatment in the past 4 weeks, (4) had no conditions that could significantly impact their QoL in the short term, and (5) had no disabilities. After clinical assessment, patients completed a form with demographic, clinical, and health status data (ASQ and Eckardt scale). Non-technical staff assisted those with poor eyesight, reading difficulties, or shaky hands, providing non-directive guidance for any questions about the questionnaire.

### Health status measures

#### Eckardt clinical symptom score.

The symptom scoring system assesses the frequency of common esophageal symptoms like weight loss, dysphagia, regurgitation, and chest pressure, with scores from 0 to 3, indicating the occurrence of symptoms never, occasionally, daily, or with meals. Scores correlate with achalasia stages: 0–1 for stage 0, 2–3 for stage I, 4–6 for stage II, and above 6 for stage III. Stage 0 and stage I generally indicate significant relief of symptoms, while intervention is considered unsuccessful for stages II and III [13,14].

#### Achalasia disease-specific HRQoL questionnaire (ASQ).

The questionnaire consists of 10 items assessing various aspects of HRQoL and includes food tolerance (item 1: 1–3 points; items 2–4: 3–8 points), dysphagia-related behavior modifications (item 5: 1–3 points), pain (item 6: 1–4 points), heartburn (item 7: 1–5 points), distress (item 8: 1–2 points), lifestyle limitation (item 9: 1–2 points), and satisfaction (item 10: 1–4 points). The total score ranges from a minimum of 10 points to a maximum of 31 points. A lower score indicates a better disease-specific HRQoL [6].

### Analysis

#### Validity assessment.

Factor analysis was conducted to evaluate the internal structure of the translated questionnaire and assess the item’s importance. Principal component analysis (PCA) with varimax rotation and explanatory factor analysis (EFA) using Bartlett’s test of sphericity helped identify dimensions linked to “disease-specific HRQoL.” The model fit was determined by the Kaiser-Meyer-Olkin (KMO) value, with ≥0.8 to ≤1.00 indicating a good fit, ≥0.7 to <0.8 indicating a satisfactory fit, ≥0.6 to <0.7 indicating an acceptable fit, and values below 0.6 are considered unacceptable [10]. PCA involved plotting eigenvalues, representing the amount of variance explained by each factor. The factors with relatively larger eigenvalues were retained up to the point where there was a break (with a value >1). The uni-dimensionality was assessed by calculating the percentage of explained variance. It is recommended that the first factor accounts for more than 20% of the total variance [15].

#### Known groups technique.

In this stage of validation, the ability of the ASQ to differentiate between different groups is assessed [16]. Specifically, in this study, the focus was on classifying patients based on disease severity using the Eckardt clinical symptom score. The ASQ questionnaire was compared between two groups: achalasia patients in clinical remission (Eckardt clinical symptom score ≤ 4) and patients not in clinical remission (Eckardt clinical symptom score > 4). To compare the two groups, a t-test for independent groups was conducted.

#### Reliability assessment.

The internal consistency reliability of both the total score and subscale scores of the ASQ was evaluated using Cronbach’s alpha coefficient. Cronbach’s alpha values were used to assess the internal consistency reliability of each scale. An excellent level of internal consistency reliability is indicated by Cronbach’s alpha ≥ 0.9, a strong level by Cronbach’s alpha ≥ 0.8, an acceptable level by Cronbach’s alpha ≥ 0.7, and a reasonable level by Cronbach’s alpha ≥ 0.6 [17].

#### Feasibility.

The feasibility of the measure was evaluated by gathering data on two aspects. First, participants were asked to rate the level of difficulty they experienced while completing the ASQ questionnaire. The response options included “easy,” “moderate,” or “difficult.” Second, participants were asked to estimate the time it took them to complete the questionnaire, with response options ranging from less than 5 minutes, 5–15 minutes, or more than 15 minutes. By assessing these factors, we aimed to gauge the practicality and user-friendliness of the ASQ measure in terms of ease of completion and time required.

#### Statistics.

In cases where an item was missing from the ASQ or Eckardt clinical symptom score, it was replaced with the median value of the items within the corresponding subscale. However, if multiple items were missing from a subscale, the questionnaire was excluded from further analysis. Additionally, efforts were made to retain participants throughout the study to minimize dropout bias, such as providing detailed study explanations and follow-up reminders. These measures enhance the reliability and validity of the findings. Data analysis was performed using Excel and SPSS version 25.0 (SPSS Inc., Chicago, IL, USA).

## Results

A total of 52 (91.2%) out of 57 patients were included. Five patients were excluded because of insufficient command of the Urdu language and missing responses to the ASQ with more than one item. So, the questionnaires of 52 patients (27 males; median age 30 years-25 females; median age 48 years) were used for analysis (Table 1). Out of the selected respondents, 33 patients were suffering from Achalasia type I, while 26.9% and 9.6% were diagnosed with Achalasia type II and Achalasia type III, respectively.

**Table 1:** General characteristics of achalasia patients (n = 52) at baseline.

	Achalasia type I	Achalasia type II	Achalasia type III
	N (%)	N (%)	N (%)
**Gender**			
Male	20 (38.5)	4 (7.7)	3 (5.8)
Female	13 (16.7)	10 (21.4)	2 (9.5)
**Age (in years)**			
<30	7 (13.5)	5 (9.6)	1 (1.8)
30.1-40	8 (15.4)	3 (5.8)	0
40.1-50	6 (11.5)	2 (3.9)	2 (3.9)
>50	12 (23.1)	4 (7.7)	2 (3.9)
**Educational status**			
No formal education	5 (9.6)	3 (5.8)	1 (1.8)
Primary	6 (11.5)	4 (7.7)	1 (1.8)
Secondary	18 (34.6)	4 (7.7)	1 (1.8)
Tertiary	4 (7.7)	3 (5.8)	2 (3.9)
**Marital Status**			
Single	10 (21.4)	4 (7.7)	2 (3.9)
Married	20 (38.5)	9 (17.3)	3 (5.8)
Widowed	3 (5.8)	1 (1.8)	0
**Employment Status**			
Employed	17 (32.7)	3 (5.8)	1 (1.8)
Unemployed	0	1 (1.8)	1 (1.8)
Retired	1 (1.8)	0	0
Housewife	9 (17.3)	9 (17.3)	2 (3.9)
Student	6 (11.5)	1 (1.8)	1 (1.8)

The EFA factor model was adequate, with a significant Bartlett’s test of sphericity (p < 0.001), evaluating whether the correlation matrix is an identity matrix, meaning there is no correlation between variables. In this case, the rejection of the null hypothesis suggests that there is a significant relationship among the items analyzed. Also, a KMO value of 0.771 indicates that the sample has a moderately good level of adequacy for further EFA.

Table 2 presents communalities, reflecting the shared variance between items and extracted EFA factors. High communalities indicate strong variance explained by the extracted factors, while lower values suggest additional factors may be needed to account for the unexplained variance. For example, the item “Raw hard fruits and vegetables” has a high extraction communality of 0.889, indicating that 88.9% of the variance in this variable is accounted for by the extracted factor. However, items such as “Rice” (extraction communality = 0.689) and “How much do you agree with the following statement about how satisfied you are with your health concerning achalasia?” (Extraction communality = 0.653) have lower communalities, indicating that a smaller proportion of variance is explained by the extracted factors for these variables.

**Table 2:** Exploratory factor Loadings for the Urdu version of ASQ.

Item no.	Communality	Extraction
2	Raw hard fruits and vegetables	0.889
6	How often have you experienced pain when eating during the past month?	0.861
9	Has having achalasia limited your lifestyle?	0.835
8	When you sit down to eat a meal, are you bothered by how long it takes you to finish eating?	0.812
1	How much has achalasia limited the types of food you have been able to eat in the last month?	0.807
5	How often in the past month have you needed to drink water while eating to deal with food caught in your esophagus?	0.769
7	During the past month, how much of a problem for you was heartburn (a burning pain behind the lower part of the chest)?	0.721
4	Clear fluids (water, juice, coffee, tea)	0.704
3	Rice	0.689
10	How much do you agree with the following statement about how satisfied you are with your health regarding achalasia?	0.653

Extraction Method: Explanatory Component Analysis

*Achalasia-specific questionnaire

The PCA was conducted to assess the dimensionality of the Urdu-translated ASQ. These dimensions align with the theoretical framework of achalasia-related symptom burden and QoL. The findings from PCA further support the validity of the ASQ as a reliable tool for assessing HRQoL in achalasia patients. Table 3 presents the results of the PCA with three different measures: initial eigenvalues, extraction sums of squared loadings, and rotation sums of squared loadings.

**Table 3:** Eigenvalues and % explained variance after factor analysis.

Item no	Initial Eigenvalues		Extraction Sums of Squared Loadings		Rotation Sums of Squared Loadings	
	Total	% of Variance	Total	% of Variance	Total	% of Variance
1	4.216	42.158	4.216	42.158	3.589	35.886
2	1.514	15.142	1.514	15.142	2.428	24.282
3	1.168	11.683	1.168	11.683	1.882	18.815
4	0.563	5.626				
5	0.463	4.627				
6	0.334	3.343				
7	0.264	2.637				
8	0.211	2.115				
9	0.176	1.755				
10	0.091	0.915				

Extraction Method: Principal Component Analysis

The initial eigenvalues indicate the variance explained by each item before any rotation or adjustment. For example, the first item has an initial eigenvalue of 4.216, explaining 42.158% of the total variance. Subsequent items have progressively smaller eigenvalues, indicating a decreasing proportion of explained variance. The extraction sums of squared loadings show the variance explained by each item after the extraction process, with the same pattern as the initial eigenvalues. The rotation sums of squared loadings represent the variance explained by each item after an optional rotation process. It’s important to note that the remaining items from the fourth to the tenth do not have values reported in the table, suggesting their minimal contribution to the total variance.

Table 4 displays the Component Matrix, revealing the correlations between the items and the extracted components in the factor analysis. It helps understand the relationship of each item with the underlying factors. In Component 1, variables such as “How much has achalasia limited the types of food you have been able to eat in the last month?” (0.838), “Rice” (0.832), “Clear fluids (water, juice, coffee, tea)” (0.763) and “Raw hard fruits and vegetables” (0.713), show positive correlations suggesting that Component 1 is associated with food limitations and difficulties in consuming specific items due to achalasia. Component 2 exhibits positive correlations with variables linked to the impact of achalasia on lifestyle, including mealtime duration and daily activity limitations. Component 3 shows positive correlations with variables concerning the presence of pain and heartburn symptoms during eating, such as “How often have you experienced pain when eating during the past month?” (0.739) and “During the past month, how much of a problem for you was heartburn?” (0.590). Negative correlations indicate an inverse relationship between the variable and the corresponding component. For example, “When you sit down to eat a meal, are you bothered by how long it takes you to finish eating?” (−0.681) exhibits a negative correlation with Component 1, implying that individuals who are less bothered by mealtime duration tend to have fewer food limitations due to achalasia.

**Table 4:** The correlations between the items and the extracted components in the factor analysis by Component Matrix^a^.

Item no		Component		
		1	2	3
1	How much has achalasia limited the types of food you have been able to eat in the last month?	0.838	0.128	−0.313
2	Raw hard fruits and vegetables	0.713	0.611	−0.080
3	Rice	0.832	0.000	−0.073
4	Clear fluids (water, juice, coffee, tea)	0.763	0.376	−0.124
5	How often in the past month have you needed to drink water while eating to deal with food caught in your esophagus?	0.854	0.096	−0.224
6	How often have you experienced pain when eating during the past month?	0.555	0.080	0.739
7	During the past month, how much of a problem for you was heartburn (a burning pain behind the lower part of the chest)?	0.649	−0.005	0.590
8	When you sit down to eat a meal, are you bothered by how long it takes you to finish eating?	−0.681	0.516	0.302
9	Has having achalasia limited your lifestyle?	−0.473	0.780	−0.039
10	How much do you agree with the following statement about how satisfied you are with your health about achalasia?	−0.764	0.304	−0.078

Extraction Method: Principal Component Analysis.

^a^3 components extracted.

The Rotated Component Matrix (Table 5) clarifies variable relationships with rotated components, enhancing interpretability. Component 1 has high loadings for items related to food limitations due to achalasia, such as “Raw hard fruits and vegetables” (0.894) and “Rice” (0.658). Component 2 reflects the impact of achalasia on lifestyle and mealtime duration, showing high loadings with items like “Has having achalasia limited your lifestyle?” (0.891). Component 3 indicates pain and heartburn symptoms associated with eating, with strong loadings on “How often have you experienced pain when eating?” (0.907) and “How much of a problem was heartburn?” (0.806). The rotated component matrix helps simplify interpretation by aligning items with their most salient components.

**Table 5:** The correlations between the items and the rotated components in the factor analysis by Rotated Component Matrix^a^.

Item no		Component		
		1	2	3
1	How much has achalasia limited the types of food you have been able to eat in the last month?	0.822	−0.367	0.076
2	Raw hard fruits and vegetables	0.894	0.147	0.261
3	Rice	0.658	−0.429	0.283
4	Clear fluids (water, juice, coffee, tea)	0.824	−0.086	0.228
5	How often in the past month have you needed to drink water while eating to deal with food caught in your esophagus?	0.784	−0.386	0.160
6	How often have you experienced pain when eating during the past month?	0.180	−0.077	0.907
7	During the past month, how much of a problem for you was heartburn (a burning pain behind the lower part of the chest)?	0.263	−0.223	0.806
8	When you sit down to eat a meal, are you bothered by how long it takes you to finish eating?	−0.359	0.831	0.016
9	Has having achalasia limited your lifestyle?	0.069	0.891	−0.191
10	How much do you agree with the following statement about how satisfied you are with your health about achalasia?	−0.389	0.626	−0.375

Extraction Method: Principal Component Analysis.

Rotation Method: Varimax with Kaiser Normalization.

^a^Rotation converged in 5 iterations.

Interviews revealed that 82.7% of patients were newly diagnosed with any type of achalasia, with an Eckardt score above 4, while 17.3% (9 patients) had previously been diagnosed and were receiving follow-up care, with their Eckardt clinical symptom score being less than or equal to 4. Patients in clinical remission had lower ASQ scores, indicating higher HRQoL (mean 19.7 ± SD 8.1), compared to newly diagnosed patients with higher ASQ scores (mean 36.8 ± SD 12.2). This difference was significant (p < 0.001), highlighting the positive impact of remission on HRQoL.

To evaluate the reliability of the Urdu version of the questionnaire, Cronbach’s Alpha coefficient was computed, and the resulting value was found to be 0.89, indicating a moderate to high level of internal consistency or reliability in the measurement instrument. This suggests that the questionnaire items consistently measure the same construct.

Furthermore, a theoretical analysis was conducted to investigate the impact of removing specific questions on the overall reliability of the instrument (Table 6). Interestingly, when the question related to experiencing pain while eating food was hypothetically deleted, Cronbach’s Alpha value increased to 0.91, indicating that removing this particular question improved the internal consistency of the questionnaire. On the other hand, deleting any other question from the questionnaire led to a decrease in Cronbach’s Alpha, ranging between 0.5 and 0.75, suggesting that the remaining questions play an important role in maintaining the internal consistency and reliability of the measurement instrument.

**Table 6:** Cronbach’s alpha values if items are deleted theoretically from Urdu translated version of ASQ.

Item No		Cronbach’s Alpha if Item Deleted
1	How much has achalasia limited the types of food you have been able to eat in the last month?	0.856
2	Raw hard fruits and vegetables	0.813
3	Rice	0.772
4	Clear fluids (water, juice, coffee, tea)	0.863
5	How often in the past month have you needed to drink water while eating to deal with food caught in your esophagus?	0. 71
6	How often have you experienced pain when eating during the past month?	0.910
7	During the past month, how much of a problem for you was heartburn (a burning pain behind the lower part of the chest)?	0.682
8	When you sit down to eat a meal, are you bothered by how long it takes you to finish eating?	0.653
9	Has having achalasia limited your lifestyle?	0.626
10	How much do you agree with the following statement about how satisfied you are with your health concerning achalasia?	0.750

*Achalasia-specific questionnaire

During feasibility evaluation, 73.1% of participants (38 individuals) found the translated questionnaire easy to use, indicating clear instructions and a user-friendly format. A further 10 patients (10.2%) were managed with minimal difficulty. In comparison, only 4 patients (7.7%) found it challenging, highlighting potential areas for further refinement or clarification of certain aspects of the translated version to enhance its usability for a wider range of individuals. 82.7% of participants completed the questionnaire in under 15 minutes, suggesting conciseness. However, 9 patients required more than 15 minutes to read, comprehend, and respond, indicating a need for additional support or simpler language for some items.

## Discussion

In a review published in 2021, the use of the Eckardt score as a prominent measure for assessing achalasia outcomes was extensively discussed, highlighting the need to use the Eckardt score, which has gained widespread acceptance and recognition in the field, with its endorsement by esteemed organizations such as the International Society of Diseases of the Oesophagus and the American Society of Gastrointestinal Endoscopy [18]. Also, the RAND Delphi process aimed at establishing consensus on quality indicators for achalasia. This process involved a panel of experts who assessed various aspects of achalasia care, revealing a lack of confidence in the current patient-reported outcome measures’ ability to accurately identify treatment failure and the need for further intervention, particularly in specific populations [5,18]. This suggests that the existing measures used to evaluate achalasia outcomes can be improved, as they may not capture the full extent of treatment effectiveness and patient experiences.

So, it was aimed to validate a disease-specific HRQoL measure, i.e., ASQ in Pakistani achalasia patients speaking Urdu, a local and national language. Our results demonstrated highly acceptable reliability and validity of ASQ in Urdu-speaking Pakistani achalasia patients. As suggested by studies on HRQoL adaptation in South Asia [19], the Urdu-translated ASQ could serve as a foundational tool for similar South Asian contexts, helping to generate region-specific data and improve healthcare practices, specifically for patients suffering from rare disorders. The findings support the call for more cross-cultural research to ensure that HRQoL metrics are adaptable and globally applicable.

Our study followed a robust adaptation process validated by experts and patients, consistent with the cross-cultural adaptation standards [20]. As achalasia is a rare disorder in which symptom onset is typically gradual and progresses over years, significantly affecting QoL with treatments focused on symptom relief rather than cure [21]. Therefore, self-management of symptoms is crucial for patients, and for this purpose, they have to be aware of their current HRQoL status. Similar methodologies have been used successfully in adapting HRQoL tools for various populations, confirming that comprehensive adaptation enhances both content validity and user relevance [22].

The symptom severity and HRQoL outcomes observed here align with findings from European and North American studies [3], indicating that untreated achalasia leads to significant QoL declines regardless of geographic location. Differences in social and cultural factors, however, can influence patient perceptions and management, as observed in HRQoL studies in Middle Eastern contexts [9]. The majority of patients found the questionnaire easy to use, aligning with studies that emphasize the importance of clarity and cultural relevance in HRQoL tools [10,12]. Similar usability studies, like those conducted in China and Latin America, show that culturally adapted questionnaires improve patient comprehension and response accuracy [23], underlining the utility of carefully adapted tools for the Pakistani context.

The validated Urdu version of the ASQ holds significant potential for improving clinical practice in Pakistan. Given the limited availability of standardized symptom assessment tools in the local healthcare system, this questionnaire can serve as a reliable instrument for symptom evaluation, aiding in both diagnosis and monitoring of achalasia patients. Future research should focus on expanding the validation of the Urdu ASQ across diverse clinical settings and larger, more heterogeneous patient populations to enhance its generalizability. Its implementation in gastroenterology clinics, particularly in settings where HRM is not readily available, can enhance early symptom detection and facilitate timely referral for diagnostic confirmation. Furthermore, the availability of an Urdu ASQ allows for more comprehensive patient-reported outcome assessments, contributing to personalized treatment strategies and improved patient care.

Additionally, longitudinal studies assessing the questionnaire’s responsiveness to treatment outcomes would provide valuable insights into its utility for monitoring disease progression and therapeutic effectiveness. Further psychometric evaluations, including test-retest reliability over extended periods, could strengthen the instrument’s stability. Moreover, exploring the adaptation of ASQ for digital health applications, such as mobile-based patient symptom tracking, could improve accessibility and real-time symptom management. Moreover, studies comparing the Urdu ASQ with other achalasia-specific tools in cross-cultural contexts would provide deeper insights into its global applicability.

However, conducting the study in a single center in Pakistan may restrict the applicability of the findings to other regions within the country or globally, as healthcare resources and patient demographics could vary, including the multilingual approach of the Pakistani population. Future work could benefit from longitudinal designs and larger sample sizes to validate these findings further, especially research on HRQoL in chronic disease contexts.

## Conclusion

This study concluded that the Urdu-translated ASQ exhibits strong psychometric properties, with high internal consistency, good construct validity, and reliable factor structure, confirming its suitability for assessing achalasia symptoms in Urdu-speaking populations. The validation process ensured linguistic and cultural adaptation while maintaining the integrity of the original instrument. This study supports these findings, with participant feedback indicating that cultural nuances were largely well-reflected and underscoring the importance of continuously evaluating and refining health and QoL outcome measures in achalasia research and clinical practice by using a translated version of ASQ in achalasia patients in Pakistan. By addressing the limitations identified by the expert panel, future efforts can strive to develop more comprehensive and reliable tools for assessing treatment outcomes and guiding decision-making in achalasia management.

## Supporting information

S1 Table Achalasia specific questions. (DOCX)
