# Supplementary material for: Urdu adaptation and validation of a disease-specific quality-of-life questionnaire in a Pakistani population of Achalasia
Source: PLoS One. 2025 Apr 17;20(4):e0321933. doi: 10.1371/journal.pone.0321933 (PMC12005510; doi:10.1371/journal.pone.0321933)
Supplement: S1 Table — (DOCX) [file pone.0321933.s001.docx]

**S1 Table. Achalasia Specific Questions**

| **#** | **Questions** | **Responses** | | | | | | | | | | | | | | |
| --- | --- | --- | --- | --- | --- | --- | --- | --- | --- | --- | --- | --- | --- | --- | --- | --- |
| 1 | How much has achalasia limited the types of food you have been able to eat in the last month? | 1 (not limited at all) | | | | 2 (Somewhat limited) | | | | | | | 3 (Moderately or severely limited) | | | |
| **2** | Raw hard fruits and vegetables | 1 (Can Swallow with no Problem) | | | | 2 (Can swallow but with a little difficulty) | | | | | | | 3 (Can swallow with great difficulty or not at all) | | | |
| 3 | Rice | 1 (Can Swallow with no Problem) | | | | 2 (Can swallow but with a little difficulty) | | | | | | | 3 (Can swallow with great difficulty or not at all) | | | |
| 4 | Clear fluids (water, juice, coffee, tea) | 1 (Can Swallow with no Problem) | | | | 2 (Can swallow but with a little difficulty) | | | | | | | 3 (Can swallow with great difficulty or not at all) | | | |
| 5 | How often in the past month have you needed to drink water while eating to deal with food caught in your esophagus? | 1 (Never/Rarely) | | | | 2 (Sometimes) | | | | | | | 3 (Frequently/Every Time I Eat) | | | |
| 6 | How often have you experienced pain when eating during the past month? (Please circle one.) | 1 (never) | | | 2 (rarely) | | | | 3 (sometimes) | | | | | 4 (frequently) | | |
| 7 | During the past month, how much of a problem for you was heartburn (a burning pain behind the lower part of the chest)? (Please circle one.) | 1 (no problem) | 2 (mild problem) | | | | 3 (moderate problem) | | | | | 4 (severe problem) | | | | 5 (very severe problem) |
| 8 | When you sit down to eat a meal, are you bothered by how long it takes you to finish eating? | Yes | | | | | | | | No | | | | | | |
| 9 | Has having achalasia limited your lifestyle? (Please check one.) | Yes | | | | | | | | No | | | | | | |
| 10 | How much do you agree with the following statement about how satisfied you are with your health in regard to achalasia?  “I am satisfied with my health in regard to achalasia” | 1 (strongly agree) | | 2 (agree) | | | | 3 (neither agree or disagree) | | | 4 (disagree) | | | | 5 (strongly disagree) | |
